# An Evolutionary Perspective of Dyslexia, Stress, and Brain Network Homeostasis

**DOI:** 10.3389/fnhum.2020.575546

**Published:** 2021-01-21

**Authors:** John R. Kershner

**Affiliations:** Department of Applied Psychology, University of Toronto, Toronto, ON, Canada

**Keywords:** dyslexia, brain networks, system homeostasis, stress, evolution

## Abstract

Evolution fuels interindividual variability in neuroplasticity, reflected in brain anatomy and functional connectivity of the expanding neocortical regions subserving reading ability. Such variability is orchestrated by an evolutionarily conserved, competitive balance between epigenetic, stress-induced, and cognitive-growth gene expression programs. An evolutionary developmental model of dyslexia, suggests that prenatal and childhood subclinical stress becomes a risk factor for dyslexia when physiological adaptations to stress promoting adaptive fitness, may attenuate neuroplasticity in the brain regions recruited for reading. Stress has the potential to blunt the cognitive-growth functions of the predominantly right hemisphere Ventral and Dorsal attention networks, which are primed with high entropic levels of synaptic plasticity, and are critical for acquiring beginning reading skills. The attentional networks, in collaboration with the stress-responsive Default Mode network, modulate the entrainment and processing of the low frequency auditory oscillations (1–8 Hz) and visuospatial orienting linked etiologically to dyslexia. Thus, dyslexia may result from positive, but costly adaptations to stress system dysregulation: protective measures that reset the stress/growth balance of processing to favor the Default Mode network, compromising development of the attentional networks. Such a normal-variability conceptualization of dyslexia is at odds with the frequent assumption that dyslexia results from a neurological abnormality. To put the normal-variability model in the broader perspective of the state of the field, a traditional evolutionary account of dyslexia is presented to stimulate discussion of the scientific merits of the two approaches.

## Introduction

An evolutionary developmental understanding of early childhood challenges the assumptions that reading disability and the effects of stress are both typically associated with neurological abnormalities. Recent theoretical advances in evolutionary developmental biology (Evo-Devo) question the justifiability of applying these assumptions widely to the general population. Evo-Devo models of stress and dyslexia, showing how early stress may lead to costly but adaptive behavioral strategies in the course of growing up, suggest that stress responsivity is not abnormal or strictly dysfunctional (Ellis and Del Giudice, 2019), although stress system dysregulation may result in children struggling to acquire fluent reading skills (Kershner, 2019, 2020a). Such conditional during typical child development contradicts the view sometimes exhibited in research and clinical studies that individuals with dyslexia are suffering from a pathopsysiological condition.

From the Evo-Devo perspective, our stress system provides a common and continuously engaged preparedness cultured by evolution to ensure the survival of the species (Lupien et al., 2009). Thus, stress system surveilance and responsivity serve an essential role in daily living for the general population, and its manifestations are not limited selectively to individuals who experience traumatic events and suffer serious behavioral and health consequences (Krugers et al., 2017; Schultz et al., 2019). Stress effects range on a continuum. Mild stress and acute challenges support a level of arousal and attention required for learning and memory (McEwen, 2007). Relatively moderate stress is pivitol in safely navigating dangerous and potentially life-threatening situations in fight or flight responses. However, even acute moderate stress may impair higher cognitive functions (Elzinga and Roelofs, 2005; Roozendaal et al., 2009; van Marie et al., 2009). Negative long-lasting physiological and behavioral consequences begin with persistent anxiety, especially in ambiguous risky environments (FeldmanHall et al., 2019). And, at the more serious end of the spectrum in vulnerable individuals may lead to poor health, psychosis and externalizing behaviors (McGowen and Mathews, 2018). In any event, vigilance and individual adaptations to life’s worrysome events are coordinated by an evolutionarily conserved balance between epigenetic, stress-induced and cognitive-growth gene expression programs (de Kloet et al., 2005; Lopez-Maury et al., 2008; Peters et al., 2017; Pryluk et al., 2019). Under optimum circumstances, this balance functions cooperatively to divide the allocation of limited attentional resources between defensive behaviors, central to coping with adverse socio-emotional events, and the complex cognitive processes required for learning. Stress system resilience modulates short- and long-term alterations between the stress/growth genetic programs (Peters et al., 2017). Moreover, the dynamic trade-off between these programs is managed by top–down activation of the hypothalamic-pituitary-adrenal (HPA) system (McGowen and Mathews, 2018; Raymond et al., 2018) and the Locus coeruleus-norepinephrine (LC/NE) system (Mather et al., 2016; Glennon et al., 2019).

The significance of this to Evo-Devo and dyslexia is that the HPA and LC/NE systems are stress reactive and, within normal limits, produce considerable interindividual response variability (Krugers et al., 2017; Ellis and Del Giudice, 2019; Schultz et al., 2019). Their combined recruitment, even under mild chronic stress, has the potential to produce excessive levels of glucocorticoids, which cross the choroid plexus blood-brain barrier, and norepinephrine, which together may (1) bias the stress/cognitive growth balance in favor of stress management, and (2) upset the homeostatic balance of processing among the major brain networks involved in beginning and fluent reading (Kershner, 2020a). Recent research in dyslexia is consistent with this hypothesis. Dyslexia has been associated with the expression of stress-related genes (Zakopoulou et al., 2019) and with dysregulation of the HPA stress system (Espin et al., 2019; Huang et al., 2020). Thus, the Evo-Devo account of stress, combined with these preliminary studies, support the possibility that prenatal and early childhood adversity may be a dyslexia risk factor, and the natural outcome of an evolutionarily conserved adaptive response to stress.

Developmental dyslexia is a hereditary, neurocognitive-based learning difficulty, usually first encountered when young children are unable to easily learn beginning reading skills. Important corollaries of a normal-variability Evo-Devo perspective of dyslexia are: (1) dyslexia represents the lower ranges of a normal curve distribution of the emerging evolution of literacy in developed countries (Pennington and Bishop, 2009; Peters and Ansari, 2019); and (2) dyslexia is independent of general intellectual performance (Tanaka et al., 2011). Depending on the skill level cut-off point, dyslexia estimates range from 5% to as high as 20% (Pugh et al., 2013). However, no reliable qualitative differences in neurobiological or cognitive processes have ever been demonstrated across the levels of reading disability (e.g., Protopappas and Parrila, 2019). Therefore, we use an inclusive definition of dyslexia. Poor readers are considered to be dyslexic if they score below-grade in reading, despite having normal IQs, adequate educational opportunities, and without a history of emotional problems (cf. Zuk et al., 2019).

This paper draws on recent experimental research in neuroscience to refine the normal-variability Evo-Devo model of dyslexia (Kershner, 2020a), and to extend the scope of discussion in considering the validity of the model. The first section outlines this perspective, which focuses on the connection between stress and dyslexia, in the broader context of the medical model orientation that has framed the scope of research and perhaps had a limiting influence on educational efforts in dyslexia over the last decades. We contrast the normal-variability model against an alternative Evo-Devo theoretical account, which proposes that dyslexia is caused by a genetic abnormality in evolutionary patterns of brain rhythmicity. The second section reviews research linking dysregulation of the cortico-limbic stress axis to the brain’s attentional networks, subordinates of the right hemisphere frontoparietal network, and the neural oscillations that are entrained atypically in dyslexia. Section three defines the stress/growth balance in terms of entropy and the brain’s major networks, supporting a speculative characterization of reading disability as a homeostatic imbalance in network interactions. The last section is a summary discussion and suggestions for future research.

## Normal-Variability vs. A Traditional Evo-Devo Model

### Main Theoretical Differences Between the Two Evo-Devo Models

The normal-variability Evo-Devo conceptualization of dyslexia is fundamentally at odds with an alternative, more traditional Evo-Devo model (Jimenez-Bravo et al., 2017; Murphy and Benitez-Burraco, 2018; Benitez-Burroco and Murphy, 2019). At the core of the traditional orientation is the assertion that dyslexia results from early onset structural aberrations of neuronal migration across multiple cortical subregions (e.g., Jimenez-Bravo et al., 2017). From this array of regional developmental anomalies, the traditionally oriented Evo-Devo model of dyslexia places special emphasis on epigenetic by environmental interactions in brain evolution, which adversely affect the species-specific, evolving genes that regulate cortical migration and patterns of brain rhythms (oscillations). The model portrays evolutionary and developmental selection of phase and phase/amplitude couplings in speech as the origin of human language, and the genes involved as the primary source of deficits associated with various categorical clinical language disorders, including dyslexia. This leads to a diagnosis of dyslexia as an “oscillopathic disease.” In contrast, the normal-variability approach, with its focus on the stress/dyslexia connection, argues that evolution in child development prioritizes selection for the genes that regulate the range and timing of neuroplasticity. Neuroplasticity of specific neuronal circuitry, then, determines the successful entrainment of patterns of brain rhythms: patterns compromised in poorer readers and suspected to be stress-linked. Also see Soloduchin and Shamir (2018) for an evolutionary account of brain rhythms as secondary to the evolutionary selection of neuroplasticity.

Both models are premised on the powerful role of the environment in the epigenetic regulation of gene expression. Numerous studies support the primary role of the environment in shaping *cis*-regulatory regions of the neuro-genome, which conserve the gene’s nucleotide sequence during phylogenetic and ontogenetic development (Brun et al., 2009; Petanjek et al., 2011; Somel et al., 2011; Zhang et al., 2011). However, in our model, such gene regulation typically produces high, but normal interindividual variability in the behaviors and brain regions undergoing evolutionary expansion (i.e., reading) (Mueller et al., 2013). And, even at the lowest levels of ability, such regulatory variability does not reflect oscillopathic disease. Rather, it is the unexceptional outcome of an emerging evolutionary skill under positive selection pressure. Thus, dyslexia in the normal-variability model is a dimensional disability shaped by selection for neuroplasticity, as opposed to a categorical abnormality associated with selection for patterns of brain oscillations.

### General Model Considerations

The more traditional view of dyslexia as a medical condition has come under increasing criticism from scholars in the field (Peters and Ansari, 2019; Protopappas, 2019; Protopappas and Parrila, 2019; Guidi et al., 2018), and from researchers who design their studies in dyslexia with the understanding that reading ability varies along a normal continuum (e.g., White et al., 2019; Zuk et al., 2019). Detailed consideration of the scientific merits of the brain abnormality claim is beyond the scope of this paper. Nonetheless, some background and several counter-intuitive examples, should raise questions about the assumption of abnormality and may serve as an entry point for future more comprehensive discussions.

Research support for approaching dyslexia within the framework of a traditional medical model invariably dates back several decades to examinations of eight postmortem brains and clinical studies of adult neuropsychological patients. There can be no doubt that rare genetic variants producing neurological abnormalities may be associated with developmental reading difficulties (Czamara et al., 2011). However, such limited evidence provides no justification for inferring that these select cases are representative of the large numbers of individuals with reading difficulties, across languages and in every literate society. A national testing program of grades 4–12 in over 8,000 schools in the U.S. revealed that only 35% of the students were grade-efficient in reading (National assessment of educational progress [NAEP],, 2015). The sheer number of school-age children and young adults with reading difficulties argues against an underlying medical condition. A second line of argument calling for reconsideration of the assumption of underlying pathology comes from the critical research review by Guidi et al. (2018). Their analysis revealed that recent experimental and gene function studies failed to substantiate the link between dyslexia and a deficit in the migration of neurons in the developing neocortex, which is a major claim undergirding the abnormality assumption and the Evo-Devo model of dyslexia as a developmental aberration (Jimenez-Bravo et al., 2017). Guidi et al. (2018) concluded their review by recommending a “fresh start” in our endeavors to understand the true nature and underlying neurobiology of dyslexia.

Predictions from the two theoretical platforms differ substantially. For example, in the traditional approach, reduced patterns of neuronal activation revealed by functional imaging experiments are expected to reflect poorer performance, while increased levels reflect better performance. In the normal-variability model, evolution seeks to maintain stress/growth homeostasis to meet task demands. The release of stress hormones, interacting with local glutamate levels, are predicted to dynamically modulate neuronal response in a classic inverted U-shaped Yerkes–Dodson, arousal response curve (e.g., Devilbiss, 2019). As a result, task performance will be degraded at points far-left and far-right on the arousal-performance curve. Hypoactivation and hyperactivation can detract equally from performance, and in any event the range of response is seen as adaptive variability. The normal-variability model predicts that task performance will be maximized at a moderate level of neuronal activation.

In addition to patterns of brain activation, neuroimaging research in dyslexia has reported more or less white or gray matter volume, more or less cortical gyrification, or differences in the efficiency of signal transmission in white matter tracts (e.g., Demonet et al., 2004). Such quantitative variations are all influenced by experience and expected in the course of normal development. Moreover, during reading and on tests of phonological awareness, good and poor readers engage the same brain areas but at different levels of intensity (Ligges et al., 2010; Kovelman et al., 2012). Indeed, Mascheretti et al. (2017) tallied 26 studies in dyslexia showing hyperactive brain areas and 42 studies showing hypoactivity. These studies represent a diversity of lines of investigation and are important for their theoretical significance. However, a valid inference of brain impairment requires evidence of structural, and behaviorally significant abnormalities in neurological organization observed in large studies with representative samples. Finally, the widely recognized candidate genetic variants for dyslexia (i.e., DYXIC1, K1AA0319, DCDC2, ROBO1) may only become risk genes if the time course of their expression is altered by experience, including stressful events, again, a part of normal development (Kershner, 2019).

In summary, the belief that dyslexia is caused by a brain abnormality is clouded by counter evidence and a lack of supporting research. Nonetheless, the two Evo-Devo models, which stand in marked contrast on this issue, need to be evaluated more specifically on their own terms. The two models differ primarily on two fundamental points. In the current model, dyslexia is a normal developmental variation resulting from selection for the genes that regulate neuroplasticity which, in turn, is essential for maintaining a balance between stress protection and cognitive growth. In dyslexia, reduced stress-related plasticity of the cognitive growth side of the equation upsets this balance. In contrast, the alternative model portrays dyslexia as a neuropathological condition originating from selection during primate brain evolution of the genes regulating the brain rhythms specific to human speech and language. Dyslexia results from a deleterious gene mutation or abnormal genetic patterns of rhythmic gene expression. Both models have identified poorer entrainment of specific oscillations in the speech envelope as the final outcome of these developmental pathways, and as an etiological factor in dyslexia. However, in the normal-variability model, poor speech tracking and entrainment are a secondary manifestation of compromised neuroplasticity. And, finally, the distinguishing feature of the normal-variability model hypothesizes that poorer oscillatory entrainment stems from adaptations to stress system dysregulation.

## Brain Oscillations, Attentional Networks, and Stress System Dysregulation

### Auditory-Phonological Oscillations and Reading

Oscillations in the flow of speech output occur simultaneously over multiple time scales in fractions of a second. The auditory receptive system has to attend, select, segment, and parse relevant sensory and linguistic features from this vocal stream for assimilation, which primarily involves frequency oscillations in the delta (1–3 Hz), theta (4–8 Hz), and low gamma (typically 35–45 but can go to 90 Hz) ranges. In the normal flow of speech expression, this amplitude envelope of modulated frequencies, varying in intensity, carries linguistic information that must be phase-locked or entrained by the receiving brain and continuously recalibrated, not only for comprehension but to support attention, memory, and the phonological processes in reading (Gross et al., 2013; Murphy, 2015; Goswami, 2019). For instance, tracking, fine-tuning and resetting by the brain (entrainment) to the rhythms in the phonological elements of speech involve the delta band for consolidating the mental representations for tonal and prosodic perception, theta for syllables, and gamma for phonemes (Goswami, 2011).

Postnatally, when brain oscillations entrain to the speech envelope, neuronal excitability facilitates the structural organization of the brain circuitry that subserves these three components of phonology. Firmly establishing the phonological lexicon in memory storage in early childhood provides the neuronal basis for making rhyme and rhythm judgments, sensitivity to syllabic stress and syllables, and phonemic awareness: all required subsequently for recruiting the word/sound/meaning pathways essential for fluent reading (Goswami, 2019). Decoding written words and sentences for meaning depends on the quality of the hierarchical structure in memory of these oscillations and their phonological units. Simply stated, early entrainment sets the stage, via attentional controls, for the visual word form in beginning reading to activate the distributed representations of the oscillatory and phonological hierarchies. During this protracted period of learning, phrasal tonality comes before syllabic stress and awareness of syllables, and syllabication comes before learning the phonemic constituents of the syllables. Learning to read and read well depends on the sequential, top–down acquisition of this hierarchy, and the ability to rapidly access from memory the entire package when decoding connected text. Thus, the degree of successful phase synchronization of speech oscillations in early childhood may have a direct bearing on poor reading in light of the generally held view that poor phonological coding is a signature feature of dyslexia (e.g., Guidi et al., 2018).

We can reasonably assume an embryonic automatic period of auditory entrainment (e.g., Telkemeyer et al., 2011), but postnatally, entrainment is not stimulus driven (Gross et al., 2013; Poeppel and Assaneo, 2020). Such speech tracking or entrainment is a core ingredient of endogenous selective attention (e.g., Lakatos et al., 2008; Obleser and Kayser, 2019), and is thought to be modulated by regional cortical areas that work together (Power et al., 2016). These topologically linked and co-activated neuronal assemblies are strongly right hemisphere lateralized (Golumbic et al., 2012; Daitch et al., 2013; Szczepanski et al., 2014; Marshall et al., 2015b; Spagna et al., 2015, 2016, 2018; Poeppel and Assaneo, 2020). Of particular interest, is that the low frequency oscillations (i.e., delta, theta), which entrain the slower wide-band fundamental rhythmic components of the phonological dictionary, are under control of the right lateralized, ventral attention (VAN) and bilateral dorsal attention (DAN) networks (Gross et al., 2013).

Pronounced hemispheric asymmetries in speech tracking are supported further by the Asymmetric Sampling in Time (AST) model of speech processing (Poeppel, 2003; Giraud and Poeppel, 2012). After initial registration bilaterally in primary auditory areas, speech entrains the phase of delta/theta in the right hemisphere, and the amplitude of gamma in the left, where gamma is down-sampled by theta to match the optimum frequency for selective extraction and coding of phonemic information. Generally, gamma is committed to local left hemisphere processing in the 35–45 Hz range, while delta and theta (1–8 Hz) in the right may also drive cross-hemispheric long-distance communication. Moreover, low frequency entrainment and cross-hemisphere signaling depend on the initiation of synaptic plasticity (Hahn et al., 2019). These traveling oscillations are flexibly coupled, with the phase of the slower frequencies powering the amplitude of the faster frequencies (Zhang et al., 2018; Benitez-Burroco and Murphy, 2019). For instance, in such phase/amplitude couplings (PACS), the phase of delta oscillations can modulate the amplitude of theta, and the phase of theta, as in processing phonemes, can modulate the amplitude of gamma. Theta/gamma PACs may have from 4 to 8 cycles of gamma for every cycle of theta, and the precision of the ratio is thought to facilitate computations for specific linguistic operations (Murphy, 2015). It follows, that gamma under- or over-sampling, tied to poor quality low frequency entrainment, would result in an unusual and faulty temporal format for phonemic categories. Finally, In reading there is some evidence that theta and gamma have their origins in the hippocampus, are coupled by the thalamic reticular nucleus, and arguably forwarded by the right attentional networks across the corpus callosum (the main conduit carrying signals between hemispheres) to instantiate the excitability needed for phonemic processing by the left hemisphere dorsal reading network (Gross et al., 2013; Marshall et al., 2015a; Murphy, 2015; Molinaro et al., 2016; Meyer, 2017).

To summarize, converging evidence supports the view that oscillations dedicated to enhancing the brain’s phonological lexicon and required for reading, are powered by low frequencies, and are phase and PAC modulated by the predominantly right ventral (VAN) and dorsal (DAN) attention networks for (1) early establishment, (2) later accessibility, and (3) routing of information between networks and hemispheres.

### Attentional Networks and Auditory Entrainment in Dyslexia

The coordinated functions of the right hemisphere ventral (VAN) and bilateral dorsal (DAN) attention networks (Corbetta et al., 2008; Petersen and Posner, 2012; Daitch et al., 2013; Gross et al., 2013; Duecker and Sack, 2015; Frarrant and Uddin, 2015; Spagna et al., 2016), which play a key role in synchronizing brain oscillations (cf. Thiele and Bellgrove, 2018), evolved as a survival mechanism to bring perceptions to consciousness, and to regulate content processing networks (Petersen and Posner, 2012). VAN’s primary hubs are the right inferior frontal cortex (IFC) and right temporal parietal junction (TPJ). DAN’s primary hubs are the right frontal eye field (FEF) and bilateral intra-parietal sulci (IPS). DAN’s right hemisphere frontoparietal circuit controls multimodal orienting across the span of attention, while VAN modulates DAN’s interhemispheric rivalry between its bilateral posterior hubs interconnecting the right IPS with the left IPS (Duecker and Sack, 2015). VAN and DAN receive modality-specific inputs, and are coordinated top–down in hierarchical control by right hemisphere hubs of the salience (SN) and frontoparietal (FPN) networks, which together, form a supramodal cognitive control network (CCN) (Menon and Uddin, 2010; Spagna et al., 2015, 2018; Wu et al., 2018, 2019). Controlling hubs of the SN are the right frontal insular cortex (FIC) and the right frontal cingulate cortex (FCC). Main hubs of the FPN, which more directly modulate the attentional networks, are the right dorsolateral prefrontal cortex (DLPFC) and right posterior parietal cortex (PPC).

Thus, VAN and DAN, with delta and theta as main carrier frequencies (e.g., Gross et al., 2013) and oversight from the FPN, may modulate the entrainment and processing of the oscillations that make up the repertory of the brain’s phonological information. VAN and DAN are allied closely in their functions and richly interconnected by: (1) right posterior parietal nodes in common with the FPN (Petersen and Posner, 2012); (2) the second branch of the right superior longitudinal fasciculus (Chica et al., 2018); and (3) the right posterior middle frontal gyrus (Corbetta et al., 2008). DAN is activated by alerting signals and expectations, and motivated by current goals, directs the focus of attention, orienting, and response selection. VAN responds with DAN by gating behaviorally relevant inputs, inhibiting distractions when DAN is focused, and is a circuit breaker for reorienting and resetting attentional focus (Corbetta et al., 2008; Daitch et al., 2013).

It is a reasonable conjecture that VAN/DAN’s survival functions, nurtured by evolution in the pursuit of attentional controls, have been adaptively appropriated by selection pressure to subserve the low frequency rapid focusing, reorienting, and resetting of attention needed for (1) the entrainment of human speech and (2) literacy. In human evolution, VAN and DAN have been repurposed, but retain their primitive survival functions. A frequent view of the behavioral systems involved in VAN/DAN cooperative interaction are alerting and orienting (Fan et al., 2009; Petersen and Posner, 2012; Spagna et al., 2015). The alerting function sustains a state of arousal and readiness, while orienting selects the most relevant endogenous and exogenous events. The supramodal cognitive control network (CCN) coordinates the alerting and orienting functions, resolves conflicts among competing mental events, and organizes response selection. Hence, the VAN/DAN complex of coactivated controls is well-suited in its genetic predisposition for (1) the selective alignment of neuronal oscillations with the rhythms of incoming speech, (2) maintaining the focus of attention for sampling, and (3) continuously resetting the entrainment process to accommodate the rapid flow of the amplitude envelope.

Behavioral research has shown that individuals with dyslexia are poorer in aspects of both VAN/DAN functions (Goldfarb and Shaul, 2013; Gabay et al., 2020), and imaging studies with children and adults with dyslexia have reported weaker encoding of the delta band (Power et al., 2013, 2016; Soltesz et al., 2013). Testing for oscillations by hemisphere interactions, multiple studies have converged in identifying the right hemisphere as the source of the atypical encoding of the low auditory frequencies in dyslexia (Hamalainen et al., 2012; Lizarazu et al., 2015; Cutini et al., 2016; Molinaro et al., 2016; Di Liberato et al., 2018) (for a review, see Kershner, 2020b). The studies by Cutini et al. (2016); Power et al. (2016), and Di Liberato et al. (2018) compared children with dyslexia to age and reading-level matched groups of good readers, effectively eliminating the possibility that the low frequency anomaly may result from their poorer reading. The study by Cutini et al. (2016) localized the right hemisphere’s poorer entrainment to the supramarginal gyrus (SMG) and angular gyrus (AG), key nodes of the TPJ with common posterior parietal connectivity with both VAN and DAN. Finally, there is some evidence that individuals with dyslexia may fail to show left hemisphere dominance for phonemic processing of gamma, and may oversample gamma in the right (Lehongre et al., 2011; De Vos et al., 2017); both likely caused by poorly specified delta/theta entrainment, with delta the first priority as delta provides the fundamental underlying power within the phonological hierarchy (Meyer, 2017).

Goswami (2011, 2019) was the first to hypothesize atypical encoding by the right hemisphere of low frequency delta/theta oscillations as an etiological factor in dyslexia. Faulty VAN/DAN attentional controls as the source of the entrainment deficiency adds a new dimension of support for Goswami’s temporal sampling framework (TSF). When the brain cannot detect temporal order in the speech stream, a continuous processing mode suppresses low frequency oscillations (Lakatos et al., 2008). According to the TSF, the right hemisphere’s atypical entrainment and insensitivity to amplitude changes or resets in the low frequency range (<10 Hz) prevents consolidation of the phonological building blocks that are called upon subsequently for efficient phonemic processing by the left hemisphere. It is generally assumed that phonemic processing by the left is carried out by PAC interhemispheric transfer powered by the right. However, Di Liberato et al. (2018) has presented evidence that low frequencies also entrain phonemic features directly in the right hemisphere. Thus, the VAN/DAN right hemisphere constellation of integrative attentional controls may entrain delta, theta, and to some extent, also gamma.

In summary, a considerable pool of experimental evidence supports the hypothesis that poor modulation of the entrainment and synchronized processing of auditory low frequency oscillations by VAN and DAN may be an etiological factor in dyslexia.

### Stress system Dysregulation in Dyslexia

Assuming this to be relatively well-established, a salient issue in the dyslexia puzzle becomes the identification of the experiential and heritability circumstances that undermine the developmental integrity of the brain’s attentional networks. The current Evo-Devo model proposes that the evolutionary selection for the timing of neuroplasticity in early childhood is a key organizing biological principle in dyslexia (Kershner, 2020a). Plasticity, defined as reorganizational capabilities in neuronal circuits and behavior in response to patterns of sensory inputs, is an essential requirement for learning to read (Vandermosten et al., 2016). The Evo-Devo model suggests that neuroplasticity may be curtailed by adversity, which destabilizes stress/growth homeostasis, leading in infancy or early childhood to suppressed low frequency entrainment by the attentional networks.

Of fundamental importance to understanding the merits of an adversity/plasticity/attention networks and reading connection, the human brain is highly neotenous (Somel et al., 2009; Bufill et al., 2011; Petanjek et al., 2011; Somel et al., 2011; Liu et al., 2012; Miller et al., 2012; Somel et al., 2014; Matsuda et al., 2020). Neoteny, or developmental allochrony, refers to the prolongation of high cortical metabolism and synaptic plasticity in regional neocortical areas in humans compared to other primates. This period of extended neuroplasticity is rooted in evolutionary cortical expansion, reflecting the regions and networks that are under positive selection for ontogenetic change and less influenced by genetic factors. Precise calculations of neuroplasticity can be made regionally and at the level of individual voxels by using PET-based measurements of aerobic glycolysis (AG, non-oxidative metabolism of glucose). For this calculation, AG is inferred whenever less than 6 molecules of oxygen are consumed for each molecule of glucose. From an early peak of maximum plasticity, the neuronal metabolism of AG demonstrates a curvilinear decrease, extending into the seventh decade of life (Goyal et al., 2017). And, significantly, AG corresponds to the energy-expensive gene expression programs supporting cognitive growth (Goyal et al., 2014, 2017; Magistretti, 2016).

In addition, repeated-measurement resting state fMRI can reveal intersubject variability in connectivity within and between specific brain networks, which may reflect points of current evolutionary selection (Mueller et al., 2013). The upshot of this is that both measurements across the brain’s networks converge in finding (1) superior AG ratings (Vaishnavi et al., 2010; Blazey et al., 2018), and (2) topmost interindividual variability (Mueller et al., 2013) in the frontoparietal control and attentional networks, i.e., FPN, VAN and DAN. From this, we can conclude that in typical development the right hemisphere attentional networks, which are dominant over the left (e.g., Gross et al., 2013; Spagna et al., 2018), are poised for growth with an elevated level of neuroplasticity.

### HPA Stress System

In dyslexia, however, this plasticity appears to be compromised (e.g., Cutini et al., 2016). Two major stress-reactive neuromodulatory systems for arousal and attention are mediating candidates in dyslexia for a linkage between stress and the loss of attentional network plasticity. One is the HPA system (McGowen and Mathews, 2018; Raymond et al., 2018) and the other is the Locus coeruleus-norepinephrine (LC/NE) system (Glennon et al., 2019). Under mild stress, negative feedback loops from the HPA to the brain serve to moderate homeostatic balance, simultaneously buffering against the potential adverse effects of strong emotions while facilitating neuroplasticity in higher cortical regions (Kershner, 2020a). However, as disturbing events accumulate in number, intensity, and duration, the HPA system may go into allostatic overload (Burns et al., 2018). When that happens, stress protection becomes paramount. The HPA releases a supraoptimal flood of glucocorticoids (cortisol in humans), which can potentially have toxic neurological effects (Peters et al., 2017; McGowen and Mathews, 2018). Such stress effects have been shown to influence synaptic number, dendritic spine formation, and arbor shaping, in the hippocampus, amygdala, and prefrontal cortex (PFC). To mitigate extensive cellular metabolic damage, individuals have the innate adaptive capacity to accelerate maturation of the brain’s emotional circuits (Bath et al., 2016; Callaghan and Tottenham, 2016) and areas subserving higher cognitive processes (Gur et al., 2019). Faster development is an evolutionary strategy which counters neoteny, but may increase survival and fitness in uncertain and threatening environments (Krugers et al., 2017; Ellis and Del Giudice, 2019). Precocious maturation dampens the HPA production of cortisol, but at a cost of neuroplasticity in the expanding brain regions subserving recently evolved and evolving skills such as reading (Wagner et al., 1997; Peters et al., 2017; Gollo et al., 2018; Benitez-Burroco and Murphy, 2019). The adaptive effect on the brain’s hierarchical organization is a loss of top–down control, which offsets the stress/growth balance by reallocating neural resources from higher cognitive functions to stress guardianship (Elzinga and Roelofs, 2005; Arnsten, 2009; Qin et al., 2009; Roozendaal et al., 2009; van Marie et al., 2009; Zhang et al., 2020). At its core, the HPA system acts as a master switch regulating stress vs. growth genetic programs (e.g., de Kloet et al., 2005; Peters et al., 2017). Thus, stress-induced HPA dysregulation compromising neuroplasticity to favor stress management is one potential pathway for a stress connection to the attentional networks.

### LC/NE Stress System

The second stress system, the LC/NE system, has a more direct effect on the attention networks. It has its origin in the noradrenergic locus coeruleus, a small nucleus located in the brainstem (Glennon et al., 2019). The LC/NE system, which evolved to support defensive behaviors and integrate autonomous functions with higher cognitive functions, releases norepinephrine (NE) diffusely throughout the brainstem, midbrain, cerebellum, and neocortex (Totah et al., 2019). NE interacts with local glutamate levels, the brain’s primary excitatory neurotransmitter, to modulate arousal and states of alertness in a Yerkes–Dodson inverted U-shaped arousal-response curve. Glutamate induces synaptic plasticity, but if excessive can lead to neurodegeneration (Yan et al., 2020). On the one hand, moderate LC/NE stimulation via thalamocortical relays, increases the magnitude of neuronal gain (responsiveness) in primary visual and auditory pathways, improving the fidelity of signal transmission by increasing the signal/noise ratio (Waterhouse and Navarra, 2019). But, of equal significance, subsets of LC neurons have evolved to target and modulate the efficiency of higher-order multisensory signal transmission in the brain circuits controlling arousal, orienting, and attending to behaviorally relevant stimuli (Aston-Jones and Cohen, 2005; Totah et al., 2019).

Of particular relevance, the LC/NE system has efferent pathways that feed NE selectively to the right hemisphere VAN and DAN via right thalamocortical relays (Aston-Jones and Cohen, 2005; Corbetta et al., 2008; Grefkes et al., 2010; Petersen and Posner, 2012; Thiele and Bellgrove, 2018). Indeed, evidence suggests that the LC/NE system coactivates VAN and DAN, which become phase modulated at delta/theta rhythms in controlling the focus of attention and switching between networks (Daitch et al., 2013; Gross et al., 2013). Conditions of unexpected uncertainty can drive LC/NE tonic output to high levels, having the potential to suppress slow-wave synchrony and block network resets (Aston-Jones et al., 1997; Corbetta et al., 2008; Mather et al., 2016). Thus, stress-induced excessive LC/NE release has the potential to directly down-regulate plasticity to favor stress management and alter the efficiency of the attention networks.

Consideration of the combined activation of both stress systems provides a cohesive theoretical basis for causal linkages between stress axis dysregulation and reduced neuroplasticity of the attentional networks in dyslexia. But, aside from research showing that dyslexia is associated with stress-related genes (Zakopoulou et al., 2019), with dysregulation of the HPA (Espin et al., 2019; Huang et al., 2020), and with reduced plasticity of the attentional networks (Cutini et al., 2016), appreciation of the proposition is too new to have motivated the systematic research needed to test its validity. However, a recent study offers optimistic support for one of the model’s predictions (Elhadidy et al., 2019). BDNF is a brain-derived neurotropic factor that can influence the expression of sets of genes regulating synaptic neuroplasticity in response to stress (Gray et al., 2013; Gregorenko et al., 2016; Peters et al., 2017). BDNF is widely distributed, with concentration in areas of cortical expansion (i.e., VAN/DAN), where metabolism by aerobic glycolysis (AG) incites the expression of BDNF and plasticity genes (Magistretti, 2016). Excessive Stress reduces levels of BDNF and AG plasticity, and is associated with impaired memory consolidation (e.g., Menezes et al., 2020). Elhadidy et al. (2019) confirmed lower levels of plasma BDNF in a Canadian sample of 28 boys and 14 girls (6–12 years) with dyslexia compared to age-matched good readers. The results were strongly supportive of the hypothesis, with no overlap between groups in BDNF levels. In the dyslexic group, BDNF ranged from 0.86 to 1.34 ng/ml with a mean of 1.10. In the control group, BDNF ranged from 1.60 to 2.40 ng/ml with a mean of 2.00. The authors recommended BDNF testing as a biomarker for dyslexia. More cautiously, the results call for replications with reading-level controls, but are a significant endorsement of a relationship between stress-related, diminished neuroplasticity and dyslexia.

Finally, a remarkable characteristic of children’s developmental stress system reprogramming is that it can also result from epigenetic patterns of maternal or paternal inheritance (Roth et al., 2009; Kolb et al., 2012; Burns et al., 2018; Posner et al., 2019). Based on animal models, stress experienced by parents prior to mating has been shown to alter the behavioral and cortico-limbic functions of their offspring. Research has only skimmed the surface of the multiple factors involved. However, the epigenetic methylation status of HPA system promoter genes and BDNF have been identified as candidate pathways for mediating such transgenerational effects (Roth et al., 2009; Burns et al., 2018). A study that deserves some scrutiny in this context, shows apparent support for the inheritance of a right hemisphere attentional deficit linked to dyslexia (Thiede et al., 2019). In a speech-sound discrimination study, 44 at-risk, 8 to 9 day-old newborn infants were compared to no-risk infants, matched on gender, gestational and measurement age, and parental educational level. Hemispheric EEG recordings of mismatched responses (MMR) revealed that the at-risk infants failed to show typical right hemisphere vowel change, neural speech-sound discrimination. However, the study did not test for oscillations, and does not confirm an epigenetic pattern of inheritance, or whether parental stress was an issue. Nonetheless, it does suggest a hereditary basis for one aspect of underperformance of the attentional networks.

In summary, VAN and DAN, modulate low frequency entrainment, are brain networks characterized by protracted developmental features (i.e., neoteny), and are integral in day-to -day functions with the cortico-limbic stress systems. Stress that exceeds an individuals range of resilience has the potential to (1) suspend neuroplasticity, responding to cortisol outflows by the HPA system, in areas of high cortical expansion, i.e., the attentional networks and (2) suppress VAN/DAN neuroplasticity directly by overabundant NE release from the LC/NE system. Both systems are candidates for the stress-induced, compromised neuroplasticity that may underly the disrupted delta/theta entrainment in dyslexia. Such epigenetic, transcriptional and functional reprogramming of the stress axis may occur prenatally or in early childhood, and we have to allow for the possibility of inheritance from the preconceptual stress experienced by parents. Still absent, however, are more direct tests confirming the specific stress system(s) pathways between stress and the attentional networks.

## Neural Network Homeostatic Imbalance in Dyslexia

### Network Stress/Growth Balance

The research reviewed supports the significance of VAN and DAN, and the potential role of stress, in children’s acquisition of the discrete phonological fundamentals required to easily learn how to read. However, for a comprehensive understanding of dyslexia, how this plays out in the broader context of collaboration among the brain’s major networks may be essential (Bailey et al., 2018). Evo-Devo’s core concept of fitness as a stress/cognitive growth balance is imbedded in the brain’s network architecture, where affective reactions to stress are focused internally and processed by the default mode network (DMN), while cognitive requirements are focused externally and controlled by the frontoparietal network (FPN) and attentional networks (Dixon et al., 2017; Schultz et al., 2019).

In coordinating network adjustments to stress, a network stress/growth tradeoff is keyed by activation of the central core of the DMN. The central core is made up of the medial prefrontal cortex (MPFC), precuneus, and posterior cingulate cortex (PCC), and is an extension of the brain’s stress axis (Andrews-Hanna et al., 2010; Wu et al., 2018; Satpute and Lindquist, 2019). The DMN central core, largely independent of sensory input, is thought to engage in self-referential processing, high-level emotion, and autobiographical memory (Dixon et al., 2017).

Stress that challenges the stress/growth balance can be defined at three levels: (1) the cellular metabolic level (Halliday and Mallucci, 2019; (2) a psychological level, as uncertainty (Peters et al., 2017); and (3) in terms of energy transfer in evolution, as entropy (Pryluk et al., 2019). It is a fundamental principle in evolution, and organic playout of the 2nd law of thermodynamics, that evolution seeks to maximize the production of entropy in non-linear and non-eqilibrium biological systems (Bazarov, 1964; Jeffrey and Rovelli, 2020). A balance between stress and growth during development is such a system. Greater entropy promotes increased cognitive complexity and the flexibility of permitting selection among competing thoughts and actions (cf. Fan et al., 2014). In this context, a stress/growth balance is orchestrated within a homeostatic range by dynamic fluctuations between the lower entropy DMN (stress biased) and high entropy FPN and attentional networks (growth biased). In effect, in stress management, evolution strives to maximize plasticity by maintaining a progressive state of a system’s non-equilibrium within a range favorable to cognitive growth (Martyushev, 2013). The salient point is that stress usually functions to keep the system in relative balance, but if excessive may circumscribe entropy at all three levels, reflected in reduced plasticity of potential arrangements of the stress/growth system. Stress, depending on the source, duration and intensity, may (1) lower plasma BDNF, (2) limit the strategies available in coping with uncertainty, and (3) reduce the complexity of high-level cognitive reasoning. Supraoptimal stress reallocates metabolic, behavioral, and energy resources from growth to the more primitive, stable side of the equation.

### The Significance of Negative Network Connectivity

The CCN (i.e., cognitive control network which combines the salience and frontoparietal networks) and the attentional networks, which work together in reading (Horowitz-Kraus et al., 2015; Ihnen et al., 2015; Freedman et al., 2020), are typically anti-correlated with the default mode network (DMN) (Qin et al., 2009; Spreng et al., 2012; Dwyer et al., 2014; Fan et al., 2014; Utevsky et al., 2014; Dixon et al., 2017, 2018; Wu et al., 2018; Hugdahl et al., 2019; Zhang et al., 2020). An inverse correlation between the CNN-attentional network hierarchy and the DMN is exemplary of a stress/growth dynamic balance. For example, Fan et al. (2014) found that activation of the CCN including the DAN, in parallel with deactivation of the DMN, varied parametrically as a function of uncertainty. Establishing the same point, Wu et al. (2018) reported that activation of the CCN increased and activation of the DMN decreased as a function of entropy.

Research using dynamic causal modeling with a large sample of adults (*n* = 404) helps to put this tradeoff in an operational framework (Zhou et al., 2018). Dynamic causal modeling, an at-rest imaging procedure which provides measures of efferent vs. afferent connectivity, was used to test the causal hierarchical structure of the anticorrelation between the core default mode network (DMN) and the dorsal attention network, DAN. The study revealed that the salience network (SN), was at the top of the hierarchy, directing the anticorrelation between the DMN and DAN. Under SN top–down surveilance, the stress/growth tradeoff was modulated by excitatory signals from the DMN to DAN, simultaneously with inhibitory influences from DAN to the DMN (cf. Menon and Uddin, 2010; Critchley and Harrison, 2013; Udden, 2015; Mai et al., 2019). This reciprocity creates feedforward and backward loops that serve to stabilize the low entropy DMN under stress, while nurturing and giving an edge to the entropy needed for continued cognitive growth. Thus, moderate levels of stress serve to balance the stress/growth system and entropic balance between networks. However, excessive stress has the potential to partially reverse this dynamic by subduing DAN’s bias toward increasing entropy and inhibitory controlling pathways to the self-centered DMN.

When the DMN was first recognized as a content processing rather than control network (Power et al., 2011), its negative correlations with regional cortical areas actively engaged in task performance were puzzling and not readily interpretable. However, an Evo-Devo perspective predicts an inverse relation between the stress-activated DMN and the cognitive growth networks, and characterizes the relationship as a reflection of favorable behavioral variability, and a potential index of optimally balanced competition (cf. Koyoma et al., 2010). Thus, a negative correlation between the stress and growth networks may be a manifestation of the stress-growth genetic program trade-off, signaling the fluidity of an ongoing evolutionary process among networks.

### Network Links to Reading and Dyslexia

Research by Bailey et al. (2018) aimed to determine the relative patterns of resting-state activation across the brain’s reading networks. The researchers applied the 7-cortical networks identified by Yeo et al. (2011) to a meta-analytical data base of 11,406 imaging studies. The results demonstrated that the FPN, VAN, and DAN combined to make up 56% of the brain’s reading network activations. Thus, a large proportion of the reading brain’s activation at rest is attributable to executive control and attentional networks. These core controlling networks are thought to be anatomically separate from the traditional reading regions recruited for processing cognitive content (Petersen and Posner, 2012; Ihnen et al., 2015). Therefore, substandard development of the brain’s attentional controls would necessarily deflate the growth side of the stress/growth balance, with deleterious consequences for both reading acquisition and later reading fluency. More specifically, a stress-induced allostatic overload favoring the DMN side of the equation has the potential for negative effects at every phase of reading skill development by overriding the processing control and growth functions of the CCN, VAN, and DAN.

The VAN/DAN complex as an origin of reading difficulties is consistent with the causal link between atypical low frequency speech encoding by the right hemisphere in dyslexia (e.g., Goswami, 2019), but it is also consistent with the acknowledged role that selective attention plays in the superior visuospatial processing ability of better readers (Zhao et al., 2011; Franceschini et al., 2012; Gabrieli and Norton, 2012; Vogel et al., 2012; White et al., 2019).

The visuospatial processes with relevance to dyslexia involve the magnocellular-dorsal stream (MDS). Multiple studies have found evidence linking dyslexia to the MDS, visuospatial orienting, and the right posterior parietal cortex (e.g., Facoetti et al., 2001; Franceschini et al., 2018; Fu et al., 2018; Vidyasagar, 2019; Archer et al., 2020). The MDS or “where” stream relays retinal signals, via the lateral geniculate nucleus and along secondary visual pathways, to the right hemisphere dorsal attention network (DAN), which leads in top-down feedback control of visuospatial processing (Underleider and Mishkin, 1982; Grefkes et al., 2010). Projecting downstream, DAN (right frontal eye field and bilateral intraparietal sulci) is the interhemispheric controlling network in the MDS, with its posterior parietal axons capable of modulating gamma in the left hemisphere (Marshall et al., 2015a, b). Evidence suggests that early stages of reading are associated with increased connectivity from the left posterior parietal cortex to the visual word form area (VWFA) for both graphemic and phonological processing (Desroaches et al., 2010; Centanni et al., 2019; Moulton et al., 2019). The VWFA is a region in the ventral occipital cortex of the left hemisphere, thought to become finely tuned for reading whole words. Thus, in reading, the temporal flow of signals from the MDS and auditory pathways may be regulated by VAN/DAN, and feedforward by phase amplitude couplings (PACs) to the VWFA for word recognition and letter to sound correspondence. This invites the hypothesis that the hypoactivation of the VWFA documented in poor readers (Centanni et al., 2018a, 2019) may result from an impairment in the right attentional networks. More specifically, such a deficit, possibly resulting from stress-linked NE release to VAN/DAN, would disrupt downstream auditory and visual signal transmission, suggesting VAN and DAN as a common multisensory origin of the auditory-phonological and visuospatial deficits in dyslexia (cf. Facoetti et al., 2008; Gori and Facoetti, 2015).

The putative central role of the attention networks in dyslexia provides a blueprint for a more comprehensive model of reading and dyslexia composed of widely separated patterns of connectivity across distributed brain networks, involving the CCN, VAN/DAN, and the DMN. For instance, such a model suggests that when reading connected text for comprehension, top–down control by the CCN may modulate continuous information transactions in thought between the DMN and the attentional networks. The internally focused DMN would be tasked with extracting the emotional tone of events as they interact with our autobiographical memory and self-image, while the externally focused attentional networks assimilate the narrative flow of information in the text. However, excessive stress or a dysregulated stress axis would reset this processing balance, curtailing VAN/DAN’s negative inhibitory controls over the DMN and overengaging the DMN. Indeed, research has shown that subclinical stress may cause the DMN to disengage from the executive networks (Schultz et al., 2019). Similarly, acute stress in healthy individuals has been shown to produce large-scale network reconfigurations resulting in reduced activation of the FPN coupled with greater within network connectivity of the DMN (Qin et al., 2009; Zhang et al., 2020). When such an imbalance in processing favoring the DMN happens, individuals may become self-absorbed and unable to access the attentional and cognitive resources needed to sustain a favorable balance of the stress/growth equation. Some support for the applicability of this scenario to dyslexia comes from studies showing that better readers exhibit a negative correlation between the DMN and bilateral reading areas (Koyoma et al., 2010, 2011), while poorer readers have shown stronger connectivity between the DMN and reading-related areas (Schurz et al., 2015). Unfortunately, there is a paucity of network research in dyslexia, and none of the studies to date have included controls over reading-level. As a result, the proposed network model stands as a theoretical proposal, pending future research to examine its validity.

To summarize, the evolutionarily conserved balance between the stress/cognitive growth genetic programs is embedded in the architecture of the brain’s major attentional control and emotional networks. Maintaining a favorable range in the homeostatic competition between these networks, i.e., VAN, DAN, and the DMN, appears to be critical to both the phonological and visuospatial processing requirements of reading. Stress, defined in physiological, psychological or entropy terms, can challenge the growth side of this equation by overengaging the DMN at the expense of the functional integrity of VAN and DAN. When network homeostasis fails, evolution favors stress management. Finally, while a stress/growth balance impacting neuroplasticity is a well-established principle in Evo-Devo, firming up its applicabilty to an interactional network understanding of dyslexia will require more direct experimental evidence.

## Discussion

The research reviewed and theoretical analysis suggest the need for a reconsideration of the nature of dyslexia. The current Evo-Devo normal-variability model stands in contradistinction to the generalization that individuals diagnosed as dyslexic are suffering from an underlying, pathophysiological condition. The traditional Evo-Devo model holds to this notion, and has proposed an account of dyslexia as the result of patterns of evolutionary genetic inheritance that cause brain abnormalities. However, It is always possible that both models are correct. Each may account for a different proportion of afflicted individuals. To elaborate on this point, the normal-variability model is not a universal theory of dyslexia, and should augment, not replace extant main-stream neurobiological and cognitive theories. The current model proposes that normal variability in stress responsivity may be one of many risk factors, and consequently, it will take large-scale epidemiological studies to determine the percentage of the population whose reading impairment is stress-related. At this point in time, pending future research motivated by both Evo-Devo perspectives, neither model has the confirming experimental evidence required to move forward with reliable avenues of early diagnosis or proven strategies of early intervention and classroom remediation.

Nonetheless, there is no doubt that the two models could not be more at odds in how dyslexia should be characterized, diagnosed, and managed. The distinction between viewing dyslexia as a normal variation in the distribution of reading skills in the general population, as opposed to a disease can have a deep and lasting influence on the self-concept of those who carry the burden of this disability and their families. Knowledge of the research on causation is probably not readily available or even an issue of high importance to teachers and parents in the early school years. Children’s welfare and remediation should be our first and foremost concerns. However, when parents or their children who have been diagnosed as dyslexic are referred to a neurologist, the implication itself of neural damage oftentimes becomes a lifetime burden. In presenting an argument in favor of the Evo-Devo normal-variability model, this paper is also an attempt to serve as an entry point to stimulate a larger discussion about the scientific merits of characterizing dyslexia as a neurological abnormality.

The central organizing concept of the current Evo-Devo model is the adaptive fitness value of maintaining a favorable range of neuronal activation in the competitive, entropic balance between stress and cognitive growth for acquiring beginning reading skills and for fluent reading. More generally, an optimal range ensures complimentary interactions between stress axis surveillance and learning ability as we go about our daily lives. However, an out of balance, reprogrammed stress axis caused by excessive duress, uncertainty, and risk may encourage emotionally driven avoidance and behavioral coping strategies, but at the cost of reduced neuroplasticity of the brain’s attentional networks. Large sample studies have reported associations of low socio-economic status (SES) with underdevelopment of the regional brain structures supporting language, reading, executive, and spatial skills (Noble et al., 2006; Brito et al., 2017). This suggests that low SES may be a factor in the dyslexia/stress connection. However, a variety of stressful circumstances are encountered by all of us in everyday living. Stressors that have the potential to overload the stress axis are actually commonplace. According to surveys in the United States and Europe, one half of all adults have experienced at least one form of early adversity (Felitti et al., 1998; Bellis et al., 2014). One can imagine a higher figure were the survey taken in today’s socio-political and pandemic-ridden environment. These studies included moderate to severe early trauma caused by emotional abuse; neglect; environmental disaster; parental separation; bullying; and witnessing violence, death, or mental illness. Each of these possibilities warrant inclusion in research protocols as potential sources of the stress response leading to dyslexia. Moreover, such adaptations to dysregulation, by blunting stress axis responsivity and operating below the level of emotional disturbance, also act as a protective measure against more serious neurological, behavioral and health issues. Thus, reading disability may be a positive but costly adaptation to stress that varies along a normal continuum of stress/cognitive growth homeostasis in otherwise healthy individuals.

The insight that challenges to the stress/growth balance during development may lead to behavioral advantages as well as disadvantages forms the intellectual energy of the comprehensive Evo-Devo theory of stress proposed by Ellis and Del Giudice (2019). The current Evo-Devo model is the first to apply this universal biological principal to the ongoing evolution of literacy and reading disability. The research reviewed found substantial evidence that faulty development of the attentional networks, i.e., VAN and DAN, under the top–down control of the CCN, may be at the etiological core of the well-established auditory-phonological and visuospatial deficits in dyslexia. Drawing the link of this attentional networking failure to stress is on less secure grounds. We do not have research in dyslexia that has been motivated specifically by the hypothesis of a stress/growth imbalance. Nonetheless, the research reviewed provides indirect evidence of a stress connection, and unambiguous theoretical pathways from stress-induced dysregulation of the HPA and LC/NE systems, to the attentional networks and dyslexia. The theoretical linkage is compelling, but we need hypothesis generated research to directly address the issue.

The model makes novel predictions. A stress-linked VAN/DAN impairment is a specific and prominent feature of the model. The HPA and LC/NE systems are established psychological stressors with diffuse effects throughout the brain on arousal and neuroplasticity. Stress releases CRF (corticotrophic-releasing factor) which activates both systems, followed by a suppression of dorsal thalamic nuclei and reduced plasticity in cortical circuits (Aston-Jones and Cohen, 2005). Although several studies with dyslexia samples have observed high cortisol (HPA curtailed plasticity) and low BDNF (signaling reduced plasticity from both systems), the interaction between the HPA and LC/NE systems in dyslexia is unknown. Both warrant longitudinal monitoring in future studies. Both systems respond to environmental events to maintain the internal homeostasis necessary for survival. It is only recently, however, that LC output has been shown to produce dysfunctions in targeted high-level neural circuits. This has shifted the research priority in LC to understanding its role in higher cognitive processes. For instance, dysregulation in adrenergically stimulated sensory pathways has been associated with common neuropsychiatric disorders, i.e., ADHD, PTSD, depression, autism, and schizophrenia (e.g., Waterhouse and Navarra, 2019).

Therefore, the current model easily accommodates comorbidity in dyslexia, and presents a rationale for predicting that other disorders may also be linked to an impairment in the attentional networks. However, the LC/NE system is the brain’s exclusive source of noradrenaline, and the review shows how its direct feed to VAN/DAN may be specific to dyslexia. However that question turns out, a main prediction is that excessive release of NE to VAN/DAN will: (1) interfere with the early oscillatory entrainments underpinning the phonological lexicon,(2) compromise the visuospatial orienting and visual segmental processing of the MDS; and (3) culminate in disruption of grapheme to phonemic decoding by the VWFA. Thus, all predictions flow from the central proposal that dysfunctional outflows from the LC/NE system may be a root cause of dyslexia.

The model also has a broad range of interpretive power. For instance, studies have reported unstable and increased brainstem and cortical response variability to auditory and visual stimuli in dyslexia samples (Hornickel and Kraus, 2013; Perrachione et al., 2016; Hancock et al., 2017; Centanni et al., 2018b; Jaffe-Dax et al., 2018). Generally, such observations have been attributed to fluctuations in selective attention that fail to synchronize neural activity and fail to improve the signal/noise ratio. Weak selective attention increases spontaneous neural firing to repeated stimuli, and the same stimuli are encoded as different percepts. In other words, lack of attention results in reduced habituation to repetitive stimuli, producing an unstable representation and high response variability. The current model is consistent with these findings and interpretations. We would add specific predictions: sourcing the attentional problem to stress, to the right hemisphere attentional networks, and to failed delta/theta/gamma brain rhythm synchronization.

The review also presents evidence that the evolutionarily conserved stress/growth dynamic may be a fundamental feature of widely distributed network interactions between the brain’s control and attentional networks and the DMN. This broader viewpoint cautions against interpreting the attention network failure in isolation. Again, we are short of the research that will be required to test the Evo-Devo dyslexia model at that level of complexity. However, an interactional attentional network failure, within an integrative reading model, predicts poorer phonological and visuospatial performance, both debated as signature impairments in dyslexia. The primacy of a deficiency in phonological vs. visuospatial processing has been a long-standing, contested issue over the legitimacy of “phonological vs. “surface” dyslexia subtypes. Indeed, aside from implications of stress, the review theoretically implicates the attentional networks as a common neurobiological mechanism that may underly both processing difficulties.

Finally, there are suggestions for early identification of children at-risk. Measures of salivary cortisol (indexing HPA homeostasis), combined with plasma levels of BDNF activation (indexing neuroplasticity), taken in early childhood would be an effective estimate of HPA and/or LC/NE system dysregulation. BDNF is a brain-derived neurotropic factor gene, a major player in subserving cognitive growth in the developing brain. Moreover, BDNF has a pronounced influence on brain activation patterns associated with phonological processing, including bilateral fusiform gyri (Mascheretti et al., 2021). BDNF research is an emerging area of inquiry. In dyslexia, we have only a single study, but results from a sample of children (aged 6–12) produced strong support for BDNF testing as an early marker for dyslexia (Elhadidy et al., 2019). Such tests are easy to administer and appropriate for use during the postnatal period.

Testing the normal-variability Evo-Devo model can be approached along a variety of established lines of investigation. An informative approach with a long history in the study of attention is Posner’s (1980) valid vs. invalid cueing paradigm. The Posner Test is an index of the fidelity of covert attentional shifting, in which attention is focused from one location to another without eye movements. Thus, it provides a behavioral measure of the efficiency of VAN and DAN, determined by fMRI (Daitch et al., 2013). The procedure consists of the presentation of a spatial cue followed by a target in the cued position (valid) or in an unexpected position (invalid). By altering where the target appears and the cue-target delay, at short cue-target delays, valid cues lead to increased visual detection and reduced response times (RT) to the target. Following a valid cue (exogenous), subjects must orient to the expected location and maintain covert attention (endogenous) to the expected location. Following an invalid cue (exogenous), attention must be disengaged and redirected to the new location (endogenous). Operating in tandem, the attention networks are tasked with coordinating bottom–up with top–down computations. Moreover, making the task even more relevant to reading, VAN and DAN become phase modulated at delta–theta rhythms during orienting, reorienting, and internally switching between networks (Daitch et al., 2013).

At longer cue-target intervals, an unexpected finding occurs. Flexibility in attentional responding engages an inhibitory mechanism which delays returning to the previously cued location, and the cued location no longer has an RT advantage. This delay in responding to the cued location encourages openness to shifts of attention that may be needed to accommodate the possibility of novel and different locations, and is called “inhibition of return” (IOR) (Itti and Koch, 2001). A robust IOR response is widely observed and a desirable element of attentional allocation. Finally, targets can be presented left or right of center visual-fixation, which projects contralaterally to the right or left hemisphere forcing each hemisphere to reveal its response capabilities. Multiple studies in dyslexia using variations of the Posner Test have provided evidence of links between the MDS, hemispheric alerting and orienting, and an impairment in the right posterior parietal cortex (e.g., Franceschini et al., 2018; Fu et al., 2018).

Therefore, with added parameters including auditory stimuli, the Posner Test is well-suited as a platform for investigating the current model:

(1) Experiences of early childhood stress, delineated by the kind of stress, its onset, duration, and intensity should correlate with test performance, and predict later reading ability.
(2) Exceptional levels of cortisol and low BDNF, both reflecting stress axis dysregulation, should correlate with test performance and predict reading ability.
(3) Brain imaging using magnetoencephalography (MEG) during the test should show atypical activation simultaneously in the attentional networks and the VWFA.
(4) LC discharge is correlated with the ventral attention network’s P-300 target-related response (Corbetta et al., 2008); therefore EEG recordings over the right hemisphere during test performance should demonstrate suboptimal activation.
(5) LC discharge is also correlated with changes in pupil size, which suggests an additional indirect behavioral measure of a dysfunctional LC/NE system. Thus, pupil size can be used as a readout of noradrenergic modulation during the Posner Test (Totah et al., 2019).

In addition to the Posner Test, in the broader context of network interactions, the model predicts that children at-risk and individuals with dyslexia will show: (1) non-significant negative correlations between the cognitive growth networks and the DMN; (2) using dynamic causal modeling, an attenuated negative feedback loop extending from DAN to the DMN; (3) positive correlations between the DMN and reading areas, combined with non-significant correlations between the growth networks and reading areas; and (4) diminished entropy regionally affecting the CCN and attentional networks. For instance, multi-scale entropy (MSE) can estimate the complexity of electrophysiology and blood flow across multiple frequencies, yielding measures of flexible connectivity between networks (Wang et al., 2018). A common theme of each prediction is to test for a stress/neuroplasticity/reading connection.

Three pressing questions for future studies are: (1) to determine each individual’s threshold at which stress induces a physiological adaptation at the cellular metabolic level, reflecting the point at which stress becomes a risk factor for dyslexia. We expect this range of stress resilience to vary in a normal curve distribution; (2) to disentangle stress as a putative cause of dyslexia from the stress resulting from dyslexia. Stress as cause or effect would be expected to activate the same stress/growth cascade of neuronal interactions. Both possibilities have equally important implications for prevention and remediation; and (3) to pursue the intriguing possibility that the dysregulated stress systems linked to dyslexia may conform to patterns of epigenetic inheritance.

In conclusion, this review suggests that an entropic balance between stress and cognitive growth may be a universal evolutionary principle (1) biasing development toward protracted regional neuroplasticity and (2) encouraging the reciprocal transactions among the brain’s major networks required for literacy. A stress-induced challenge to this trade-off, which sparks a protective adaptive response, has the potential to compromise neuroplasticity in the brain’s hierarchical complex of cognitive control and attentional networks: predominantly right hemisphere networks that regulate the auditory-phonological and visuospatial processing linked etiologically to dyslexia. Thus, dyslexia in otherwise normal and healthy individuals, may result from positive but costly adaptations to stress axis dysregulation. However, a direct deleterious role for stress as a causal factor in dyslexia has yet to be tested experimentally. And, the specificity of how stress may impact levels of this multiple and hierarchical network organization is an open question for future theoretical and observational research.

## Author Contributions

The author confirms being the sole contributor of this work and has approved it for publication.

## Conflict of Interest

The author declares that the research was conducted in the absence of any commercial or financial relationships that could be construed as a potential conflict of interest.
